# EHMT2/G9a Inhibits Aortic Smooth Muscle Cell Death by Suppressing Autophagy Activation

**DOI:** 10.7150/ijbs.38835

**Published:** 2020-02-10

**Authors:** Tai-Qiang Chen, Nan Hu, Bo Huo, Jackson Ferdinand Masau, Xin Yi, Xiao-Xuan Zhong, Yong-Jie Chen, Xian Guo, Xue-Hai Zhu, Xiang Wei, Ding-Sheng Jiang

**Affiliations:** 1 Division of Cardiothoracic and Vascular Surgery, Tongji Hospital, Tongji Medical College, Huazhong University of Science and Technology, Wuhan 430030, China;; 2 Department of Cardiothoracic Surgery, Taihe Hospital, Hubei University of Medicine, Shiyan, Hubei 442000, P.R. China;; 3 Department of Cardiology, Renmin Hospital of Wuhan University, Wuhan 430060, China;; 4 Key Laboratory of Organ Transplantation, Ministry of Education;; 5 NHC Key Laboratory of Organ Transplantation;; 6 Key Laboratory of Organ Transplantation, Chinese Academy of Medical Sciences

**Keywords:** EHMT2/G9a, Vascular smooth muscle cells, Autophagy, SQSTM1, BECN1

## Abstract

Although EHMT2 (also known as G9a) plays a critical role in several kinds of cancers and cardiac remodeling, its function in vascular smooth muscle cells (VSMCs) remains unknown. In the present study, we revealed a novel function of EHMT2 in regulating autophagic cell death (ACD) of VSMC. Inhibition of EHMT2 by BIX01294 or knockdown of EHMT2 resulted in reduced VSMC numbers which were independent of proliferation and apoptosis. Interestingly, EHMT2 protein levels were significantly decreased in VSMCs treated with autophagic inducers. Moreover, more autophagic vacuoles and accumulated LC3II were detected in VSMCs treated with BIX01294 or lenti-shEHMT2 than their counterparts. Furthermore, we found that EHMT2 inhibited the ACD of VSMCs by suppressing autophagosome formation. Mechanistically, the pro-autophagic effect elicited by EHMT2 inhibition was associated with SQSTM1 and BECN1 overexpression. Moreover, these detrimental effects were largely nullified by SQSTM1 or BECN1 knockdown. More importantly, similar results were observed in primary human aortic VSMCs. Overall, these findings suggest that EHMT2 functions as a crucial negative regulator of ACD via decreasing SQSTM1 or BECN1 expression and that EHMT2 could be a potent therapeutic target for cardiovascular diseases (*e.g.,* aortic dissection).

## Introduction

Cardiovascular diseases (CVDs) are the primary cause of death globally, and more than 17 million people die yearly from CVDs 1. CVDs include a series of diseases affecting blood vessels, such as coronary artery disease, aortic aneurysms, cerebrovascular disease, peripheral artery disease, pulmonary hypertension and renal artery stenosis, as well as diseases affecting the heart. Vascular smooth muscle cells (VSMCs) are one of the foremost cellular types of vascular walls that regulate arterial vascular tone and maintain structural stability for blood flow together with elastic fibers 2, 3. In response to environmental changes, modulation of VSMC survival and function plays a crucial role in the development of CVDs. Aberrant VSMC function including migration and proliferation and inappropriate VSMC loss have been demonstrated to play crucial roles in the pathogenesis of many CVDs including vein graft failure^4^, atherosclerosis 5, pulmonary artery hypertension 6, post angioplasty restenosis 7, and aortic aneurysm/ dissection.^8^ However, the regulatory mechanisms, especially epigenetic mechanisms of VSMC homeostasis still remain largely unknown.

EHMT2 (also known as G9a) is a Su(var), Enhancer of Zeste, Trithorax (SET) domain-containing protein with histone lysine methyltransferase activity, which is responsible for monomethylation and dimethylation of lysine 9 on histone H3 (H3K9me1, H3K9me2).^9^ EHMT2 has been documented to be involved in development, pluripotency, cellular differentiation and cell cycle regulation. Selective inhibitors of EHMT2 such as BIX01294 and BRD4770 could effectively enhance autophagy in various tumors, such as breast cancer,^10^ colon cancer,^10^ bladder cancer,^11^ oral squamous cell carcinoma,^12^ and neuroblastoma.^13^ EHMT2 together with RFX5, HDAC2, and Sin3B was reported to form a repressive chromatin structure surrounding the collagen gene transcription start site in response to IFN-γ stimulation, which is responsible for IFN-γ induced COL1A2 repression in VSMCs. However, whether EHMT2 was involved in VSMC homeostasis and the specific mechanisms involved have not been reported previously.

Autophagy is a catabolic process that occurs in response to different forms of stress by eliminating harmful cytosolic components, such as damaged organelles and misfolded proteins, to maintain cellular homeostasis 14, 15. Emerging evidence has demonstrated that changes in autophagy in VSMCs regulate VSMC number and function and go hand in hand with CVD occurrence. Several studies have reported that activation of autophagy contributes to the proliferation of VSMCs. For example, platelet-derived growth factor (PDGF) treatment induced autophagy activation in VSMCs, which inhibited the expression of contractile proteins and upregulated the expression of synthetic markers.^16^ PDGF treatment was also associated with enhanced migration and proliferation of VSMCs 16. Likewise, rapamycin, an inducer of autophagy activation, is capable of inhibiting both cell proliferation and migration 17, 18. Consequently, due to the crucial role of rapamycin-induced autophagy in VSMC function, rapamycin-based drugs have been used in drug-eluting stents to prevent restenosis after percutaneous coronary intervention.^19^ Our previous study also demonstrated that autophagy was enhanced in the human aortic wall with aortic dissection 8. Uncontrolled autophagy activation leads to increased VSMC autophagic cell death (ACD), which is associated with VSMC loss and aortic dissection occurrence 8, 20. Autophagy was shown to be important in the development of CVDs via affecting VSMC survival and function. Since the critical role of autophagy during the pathophysiology of multiple diseases has been revealed, elucidation of the regulatory machinery of autophagy is important. In recent years, compelling research has revealed that histone methyltransferases are involved in autophagy regulation. Our previous research demonstrated that EZH2 protects VSMCs from ACD via autophagy inhibition, which is mediated by ATG5 and ATG7 expression and the MEK-ERK1/2 signaling pathway 8. Although EHMT2 plays a critical role in autophagy of cancer cells, whether EHMT2 participates in autophagy of VSMCs or even affects its survival remains unknown.

In the present study, our results revealed that EHMT2 regulates VSMC survival via controlling autophagy, which is mediated by affecting SQSTM1 and BECN1 expression. Thus, we demonstrated that EHMT2 is a primary regulatory factor for VSMC homeostasis.

## Materials & Methods

### Cell culture and treatment

Primary human aortic vascular smooth muscle cells (HAVSMCs) were cultured from the ascending aorta when they underwent heart transplantation at Tongji Hospital, Tongji Medical College, Huazhong University of Science and Technology. This study was approved by the Tongji Hospital, Tongji Medical College, Huazhong University of Science and Technology Review Board in Wuhan, China. As previously described, 21 aortic tissues were divided into pieces of approximately 1.5mm^2^ and stored in DME/F12 at 4°C. Then, the tissues were transferred into a new petri dish after removal of blood stains and connective tissue. The intima and media structures were identified under a stereo microscope, and the intimal and residual adventitial tissues were stripped with forceps. The dissected media of the vessels was then cut into small pieces (1-2 mm) and transferred to cell-culture flasks. The tissue blocks were evenly spread on the bottom of the flask with a control interval of approximately 2 mm. Then, 5 mL of DME/F12 medium supplemented with 10% FBS, 1% L-glutamine, and antibiotics were added to the flask, and the lid was loosely screwed on. The flask was placed in the incubator and stood upright for 30 minutes to allow the explant to attach to the wall of the culture flask. After 30 minutes, the culture bottle was lowered. The culture bottle was not moved for 5 days. Long spindle-shaped smooth muscle cells were observed around the tissue block in one week. After the cells grew, the medium was renewed every 3 days, and the state of the cells was closely observed. The smooth muscle cells around the tissue block were evenly distributed, and the cells were routinely passaged when the degree of cell fusion reached approximately 80%.

The rabbit aortic vascular smooth muscle cells (RAVSMCs) were cultured with DMEM/F12 (SH30023.01; HyClone) supplemented with 10% fetal bovine serum (1767839; Thermo Fisher Scientific), and 1% penicillin-streptomycin (15140-122; Thermo Fisher Scientific).

After starvation for 12 hours, RAVSMCs and HAVSMCs were treated with BIX01294 (S8006; Selleck) at different concentrations as indicated. Rapamycin (150 nM; S1039; Selleck) and chloroquine (20 μM; C6628; Sigma-Aldrich) were used to induce autophagy or to inhibit autolysosome degradation. Lactate dehydrogenase (LDH) release assay was detected by using Cytotoxicity LDH Assay Kit-WST^®^ kit (CK12; Dojindo).

### Western blot analysis

Western blotting was performed as previously reported.^22^-^24^ The antibodies used in this study including LC3II/I (#12741), ATG5 (#12994), ATG7 (#8558), ATG12 (#4180), ATG16L (#8089), BECN1 (#3495), Desmin (#5332), and Vimentin (#5741) were obtained from Cell Signaling Technology. Antibodies against histone H3 (ab1791), H3K9me1 (ab9045), H3K9me2 (ab1220), EHMT2 (ab185050), α-SMA (ab7817), SM22α (ab14106), and SQSTM1 (ab56416) were purchased from Abcam. PCNA (GTX100539), and p-Histone H3 (sc-8656-R) were obtained from Genetex and Santa Cruz, respectively.

### Flow cytometry

Flow cytometry analysis was performed as previously reported 8. Briefly, RAVSMCs (approximately 2-5 × 10^6^ cells) were collected by trypsin digestion and then washed twice with PBS. After centrifugation for 10 min, the cells were resuspended in 500 μL PBS. Subsequently, these cells were fixed with 5 mL of pre-cooled 70% ethanol overnight at 4 °C. After discarding the ethanol, the cells were washed twice with PBS. The cells were stained with 0.015 mg of PI (P4864; Sigma Aldrich) and 0.3 mg of Ribonuclease A (R5125; Sigma-Aldrich) for 2 h in darkness. For apoptosis analysis, the PE Annexin V Apoptosis Detection Kit I (559763, BD Pharmingen^TM^) was used. A BD FACS Aria™ III sorter was used to detect apoptotic cells and to analyze the cell cycle.

### Immunofluorescence assay and transmission electron microscopic analysis

Immunofluorescence staining was performed as previously described 8. The RAVSMCs and primary HAVSMCs were stained with α-SMA (ab7817, Abcam) antibody, and images were taken using an Olympus light microscope BX53 system.

The RAVSMCs were treated with 5 μM BIX01294 for 24 h, and then fixed with 2.5% cold glutaraldehyde for 30 min after washing with PBS twice. Transmission electron microscope (TEM) analysis was conducted as previously reported 8.

### EdU incorporation assay

Cell-LightTM Edu Apollo567 In Vitro kit (C10310-1, RIBOBIO) was used to perform EdU incorporation assay. Primary HAVSMCs were planted in 24-well plates at 1×10^4^ cells per well. After treatment with 5 μM of BIX01294 or DMSO for 24 h, the cells were incubated with 50 μM EdU medium (300 μl) for 2 h. Then, cells were washed three times by PBS, fixed by 4% paraformaldehyde for 30 min and incubated with 2 mg/ml glycine to neutralize paraformaldehyde. After PBS containing 0.5% Triton X-100 added to each well for 10 min, cells were incubated with 1× Apollo staining solution for 30 min. Next, 0.5% Triton X-100 PBS solution was added for 10 min again and cells were incubated with 1× Hoechest 33342 for 30 min at room temperature. After final washing by PBS, the cells were observed by fluorescence microscopy.

### ChIP-PCR

The ChIP-PCR was performed as we previously reported 21. In brief, approximately 8×10^8^ RAVSMCs per group were needed to extract genomic DNA. After starvation for 12 h, Control group cells were incubated with DMSO for 48 h and experimental group cells were incubated with BIX01294 5 μM for 48 h. RAVSMCs were fixed with 3% formaldehyde for 10 min at room temperature, incubated with 2.5 M glycine to stop the reaction and collected in cold cell lysis buffer (10 mM Tris-HCl pH 8.0, 10 mM NaCl, 0.2% Nonidet P-40). Lysis reaction was performed on ice for 10 min, and after centrifugation for 3000 rpm at 4℃, the deposits were acquired. The deposits were lysed in nuclei lysis buffer (50 mM Tris-Cl pH 8.0, 10 mM EDTA, 1% SDS) on ice for 10 min, sonicated and tested to ensure fragmentation. Chromatin quantified to 200 μg for further immunoprecipitation and mixed with IP buffer (2mM EDTA, 150mM NaCl, 20mM Tris-HCl pH = 8.1, 0.1% Triton X-100, Complete protease inhibitor cocktail). Protein A/G magnetic beads (Biotool, B23202) were washed twice with TE buffer (1 M Tris-HCl pH = 8.0, 0.5 M EDTA, PH=8.0) and three times with mix of TE and 1mg/ml BSA. Then, chromatin was precleared for IP with the 30 μl prepared beads and rotated at 4 °C for 2 h. The supernatants were transferred to new microcentrifuge tubes, and 2 μg of H3K9me1 antibody (Abcam, ab9045) or IgG (Santa Cruz, sc2025) was added to each tube and rotated overnight at 4 °C. The next day, 30 μl protein A/G magnetic beads were added into each sample, and rotation was performed for at least 2 h at 4 °C. Antibody-enriched beads were harvested with a magnetic holder and washed in the shaker sequentially for 5 min at 4 °C in 1 mL of wash buffer I (2 mM EDTA, 20 mM Tris-HCl pH = 8.0, 0.1% SDS, 1% Triton X-100, 150mM NaCl) × 1, wash buffer II (2mM EDTA, 20mM Tris-HCl pH = 8.0, 0.1% SDS, 1% Triton X-100, 500mM NaCl) × 2, and TE buffer× 2. Beads were resuspended in 100 μl elution buffer (1% SDS, 0.1M NaHCO_3_), rotated at room temperature for 30 min and step was repeated once again. The confluent supernatants were added 14 μl 5M NaCl, 3.5 μl 20 mg/ml Proteinase K and 3.5 μl 10 mg/ml RNase A (Sigma-Aldrich, R5125) for RNA digestion and formaldehyde decrosslinking at 65 °C overnight. DNA was purified with a DNA purification kit (Beyotime, D0033), and the chromatin was dissolved in 100 μl TE buffer. Then, 2 μl of DNA solution, primers, DEPC water and Hieff^®^ qPCR SYBR^®^ Green Master Mix(YEASEN, 11201ES08) were loaded per well for the real-time PCR assay. The primers used in this study are: SQSTM1-P1 forward: ACTGCTGATGAAGTTCTGGGAG, reverse: ACCTGCTTTCAGACTGGGGT; SQSTM1-P2 forward: GCCTTGGTCTCCTTCTATAACCT, reverse: ATGCAGGGCAGGGATTTCTC; BECN1-P1 forward: CTAAAGTTTGGGCGATGGCG, reverse: TTCAGTCCCCAACGCTCTTC; BECN1- P2 forward: AAGAGCGTTGGGGACTGAAC, reverse: GTCAGGGAAGGGGCTGTAAC.

### Plasmid construction

Double-strand oligonucleotides of shRNA targeting human EHMT2, SQSTM1 and BECN1 were cloned into pLKO.1 plasmids at AgeI and EcoRI restriction enzyme sites. The target sequences were as follows: shEHMT2: CGAGAGAGTTCATGGCTCTTT; shSQSTM1-1: CCTCTGGGCATTGAAGTTGAT;

shSQSTM1-2: CCGAATCTACATTAAAGAGAA;

shBECN1-1: CCCGTGGAATGGAATGAGATT;

shBECN1-2: CTCAAGTTCATGCTGACGAAT.

### Statistical analysis

In our present study, all the data are shown as the mean ± standard deviation (SD). Student's two-tailed *t* test was used to compare the means of two groups, while multiple group comparisons were conducted by using oneway ANOVA with least significant difference (equal variances assumed) or Tamhane T2 (equal variances not assumed) tests in SPSS software (version 13.0). *p*<0.05 was considered statistically significant.

## Results

### Inhibition of EHMT2 by BIX01294 impeded RAVSMC growth

**Figure 1:**
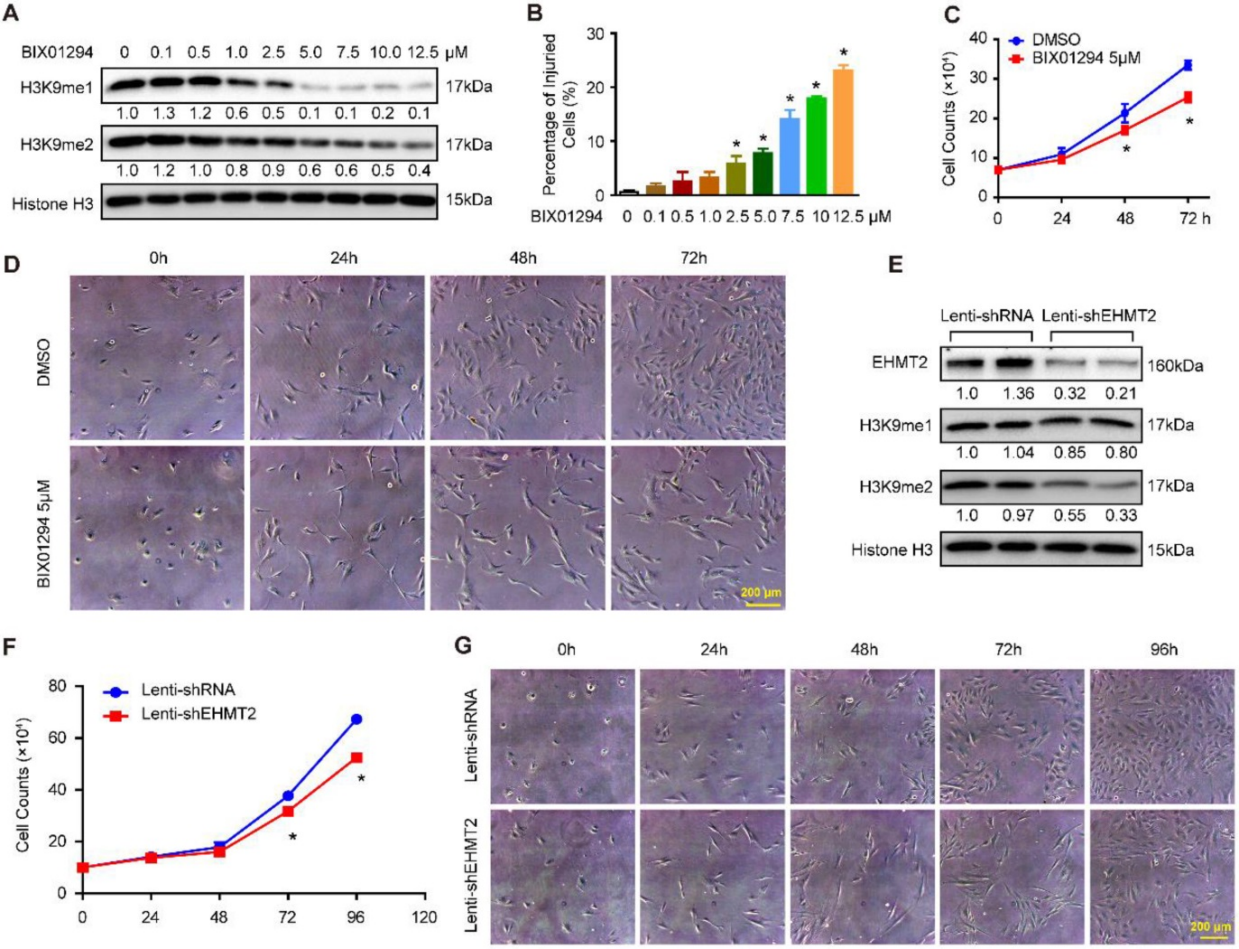
** EHMT2 inhibition or knockdown suppresses RAVSMCs growth. A.** The representative western blots of H3K9me1, H3K9me2, and histone H3 in RAVSMCs treated with different concentrations of BIX01294 for 24h (n=4 samples), histone H3 serves as a loading control. **B.** Cell injury was evaluated by LDH release after treated with BIX01294 or DMSO for 24h (n=6). *p<0.05 vs. DMSO. **C.** Cell numbers were counted at indicated time points after treated with 5 μM of BIX01294 or DMSO (n=3). *p<0.05 vs. DMSO. **D.** The representative cell images under optical microscope at different time points after BIX01294 (5 μM) or DMSO treatment (n=4). **E.** EHMT2, H3K9me1, H3K9me2, and histone H3 protein level were detected by western blot in RAVSMCs infected with lenti-shRNA or lenti-EHMT2 (n=4), histone H3 serves as a loading control. **F.** The growth curve of RAVSMCs treated with lenti-shRNA or lenti-EHMT2 for indicated times (n=3). *p<0.05 vs. lenti-shRNA. **G.** Representative images of RAVSMCs under optical microscopy (n=4).

To explore the effects of EHMT2 on VSMCs, BIX01294, an inhibitor of EHMT2, was used. First, we treated RAVSMCs with different concentrations of BIX01294 to ascertain the best dosage. Our results revealed that 5 μM of BIX01294 was the optimum experimental condition for effective inhibition of H3K9me1/2 and minor cell injuries evaluated by LDH release assay (Figure 1A and 1B). Importantly, 5 μM of BIX01294 stimuli resulted in visibly reduced numbers of RAVSMCs after 48 and 72 hours, as evidenced by the growth curve and cell images (Figure 1C and 1D). Cell cycle and apoptosis, the primary factors affecting cell numbers, were analyzed via flow cytometry. Unexpectedly, compared with DMSO treatment, no obvious difference was observed in the cell cycle and in early and late apoptosis after BIX01294 treatment (Figure S1A-S1C). Furthermore, the comparable protein levels of proliferation and contractile-related markers (including PCNA, p-H3, α-SMA, SM22, Desmin, and Vimentin) between the EHMT2-inhibited and control groups indicated that EHMT2 had no effect on RAVSMC phenotype switching (Figure S1D and S1E). Thus, these results indicated that BIX01294 inhibits RAVSMC growth independent of cell proliferation and apoptosis.

### EHMT2 knockdown inhibited RAVSMC growth

The aforementioned results indicated that EHMT2 was associated with RAVSMC growth. To further investigate the function of EHMT2 in RAVSMCs, we knocked down EHMT2 in RAVSMCs via lentivirus with short hairpin RNA (shRNA). The EHMT2 expression level and methylation level of H3K9 were verified by Western blots, and the results showed that the EHMT2 protein level and H3K9me2 were significantly reduced in RAVSMCs after shEHMT2 lentivirus infection (Figure 1E). In accordance with the results in the EHMT2-inhibited group, the RAVSMC number was also noticeably reduced after lenti-shEHMT2 infection for the indicated times (Figure 1F and 1G). Furthermore, similar cell percentages were observed both in G0/G1 and S and in G2/M cell phases with or without EHMT2 knockdown (Figure S1F and S1G). In addition, similar rate of apoptosis was detected in lenti-shRNA- and lenti-shEHMT2-infected VSMCs (Figure S1H).

### Autophagy enhancement was accompanied by decreased EHMT2 expression

**Figure 2:**
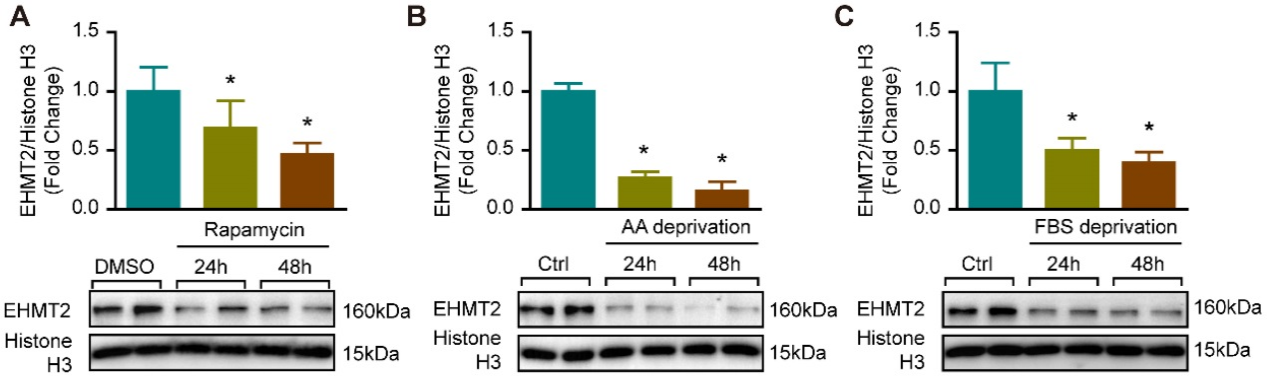
** EHMT2 protein level was reduced under autophagy related stimulation. A.** EHMT2 and histone H3 protein levels were detected by western blot in RAVSMCs treated with 150 nM of rapamycin for 24h or 48h (n=4). *p<0.05 vs. DMSO. **B.** Western blot was used to evaluate the protein levels of EHMT2 and histone H3 in RAVSMCs under amino acid (AA) deprivation or not condition (n=4). *p<0.05 vs. control. **C.** The protein levels of EHMT2 and histone H3 were assessed by western blot in RAVSMCs stimulated with FBS deprivation or not (n=4). *p<0.05 vs. control. Histone H3 serves as a loading control.

Since EHMT2 inhibition or knockdown impeded RAVSMC growth in a cell proliferation and apoptosis-independent manner (Figure S1), we curious about whether autophagy is mediated the effects of EHMT2 in VSMC. It is reported that EHMT2 plays a critical role during autophagy in cancer cells 11, 12, 25. Our previous study proved that excessive autophagic activation in VSMCs results in autophagic cell death and inhibition of cell growth 8. Thus, we verified whether EHMT2 is associated with autophagy in VSMC via an established autophagy-activated cell model by rapamycin stimulation and nutrition deprivation. Our results showed that in cultured RAVSMCs, EHMT2 protein levels were remarkably decreased after 150 nM of rapamycin treatment for 24 h and 48 h (Figure 2A). In RAVSMCs challenged with medium depriving amino acid or fetal bovine serum, the protein levels of EHMT2 also strikingly decreased when compared with normal condition (Figure 2B and 2C). These results indicated that EHMT2 may play an essential role in autophagy regulation in VSMC.

### Loss of EHMT2 function enhances autophagic activity

**Figure 3:**
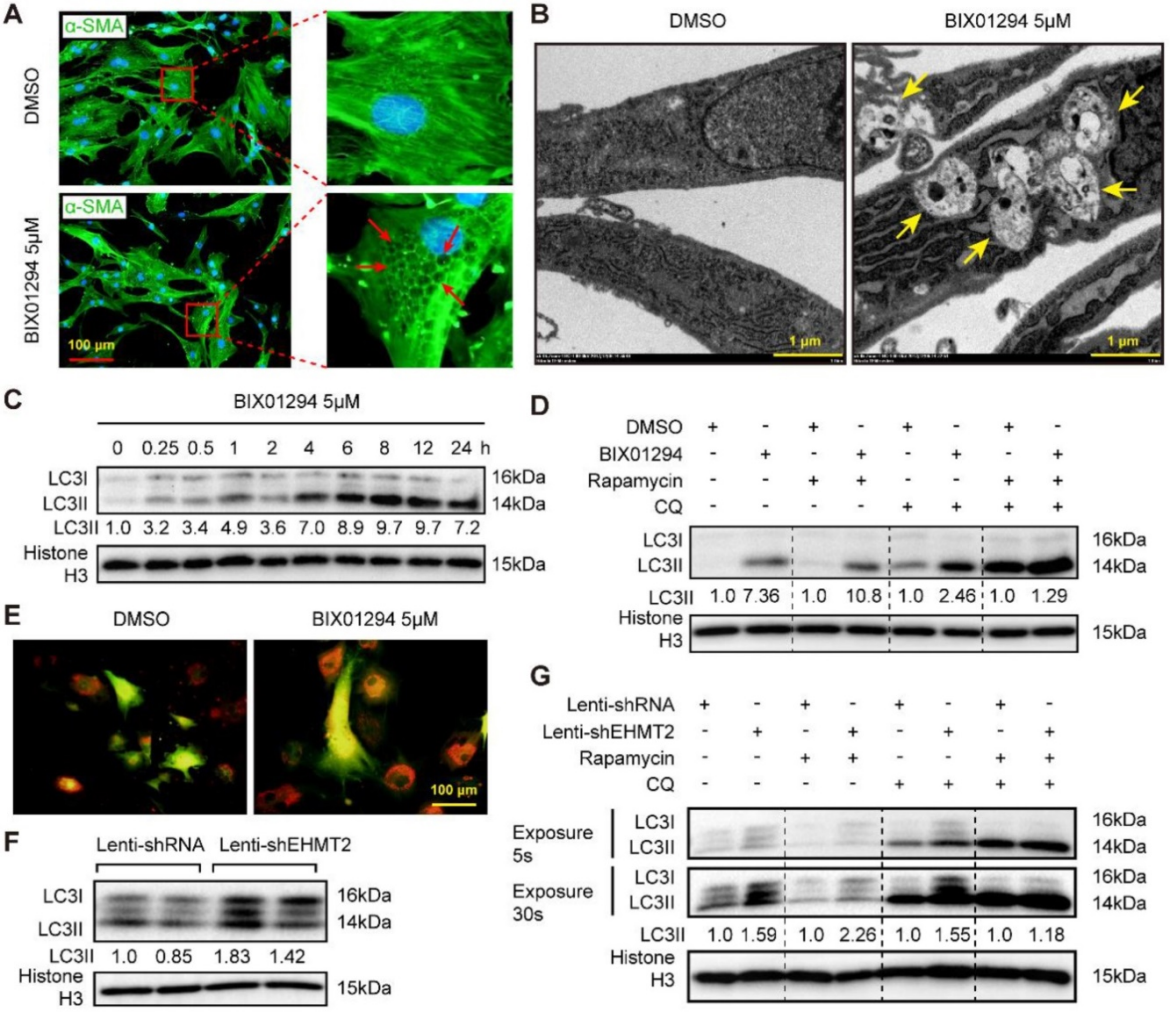
** EHMT2 inhibition or knockdown accelerates RAVSMCs autophagy. A.** The RAVSMCs were fluorescence staining with α-SMA after treated with 5 μM of BIX01294 or DMSO for 48h (n=3), red arrows indicated vacuoles in VSMCs. **B.** The representative images of autophagic vacuole under a transmission electron microscope and yellow arrow indicates autophagic vacuole in RAVSMCs with 5 μM of BIX01294 or DMSO stimulation for 12h (scale bar, 1 μm). **C.** LC3 and histone H3 protein levels were detected by western blot in RAVSMCs treated with 5 μM of BIX01294 for different times (n=4). **D.** The protein levels of LC3 and histone H3 were assessed by western blot in RAVSMCs treated with indicated stimulations (n=4). **E.** The mCherry-GFP-LC3 was overexpressed in the RAVSMCs, which were subsequently stimulated with 5 μM of BIX01294 or DMSO for 6 hours. Yellow and red indicate autophagosomes or autolysosomes, respectively (n=3). **F.** The protein levels of LC3 and histone H3 were assessed by western blot in RAVSMCs infected with lenti-shRNA or lenti-shEHMT2 (n=4). **G.** Western blot was used to evaluate the protein levels of LC3 and histone H3 in RAVSMCs treated with indicated stimulations for 8h (n=4). Histone H3 serves as a loading control.

Reduced EHMT2 expression levels during autophagy activation indicated that EHMT2 may inhibit this biological process. Interestingly, many vacuoles were observed in the cytoplasm of EHMT2-inhibited RAVSMCs, as evidenced by cell images of the DMSO or BIX01294 stimulation groups marked by α-SMA (Figure 3A). Electron microscopy was used to detect the specific structure of these vacuoles. Double-membraned vacuoles containing cytoplasmic components, namely, autophagic vacuoles, were found in EHMT2-inhibited RAVSMCs, while they were barely found in the control group (Figure 3B). Likewise, western blots showed that LC3II levels remarkably increased in the EHMT2-inhibited group as early as 15 min after BIX01294 treatment (Figure 3C). Furthermore, chloroquine (CQ, blocking degradation of autophagosome components) and rapamycin were used to test the role of EHMT2 in autophagic flux. Compared with their counterparts, CQ treatment resulted in LC3II accumulation in EHMT2-inhibited RAVSMCs (Figure 3D). CQ combined with rapamycin treatment led to further LC3II accumulation, but BIX01294 treatment has no obvious additional effects on LC3II accumulation in this condition (Figure 3D). We next infected RAVSMCs with a lenti-mCherry-EGFP-LC3 reporter plasmid to further address the effects of EHMT2 on autophagic flux. The mCherry-EGFP-LC3 system is characterized by autophagosomes marked by both EGFP and mCherry, while only mCherry exists in autolysosomes. As shown in Figure 3E, abundant red spots (autolysosomes) were observed in EHMT2-inhibited RAVSMCs, which indicated that EHMT2 inhibition resulted in more autolysosome formation and that the increased LC3II was not due to autophagic flux blockade. To further confirm these results, we tested the LC3 level in EHMT2 knockdown RAVSMCs and the control group. The results demonstrated that LC3II was drastically increased in EHMT2 knockdown RAVSMCs (Figure 3F). We also used CQ alone or CQ combined with rapamycin to stimulate EHMT2 knockdown RAVSMCs and their counterparts. Accordingly, CQ alone treatment led to more LC3II accumulation in EHMT2 knockdown RAVSMCs (Figure 3G). These results suggested that inhibition of EHMT2 enhances autophagic activation and autophagosome formation rather than affecting autophagic flux.

### EHMT2 regulates autophagy by affecting SQSTM1 and BECN1 expression to control ACD of RAVSMCs

**Figure 4:**
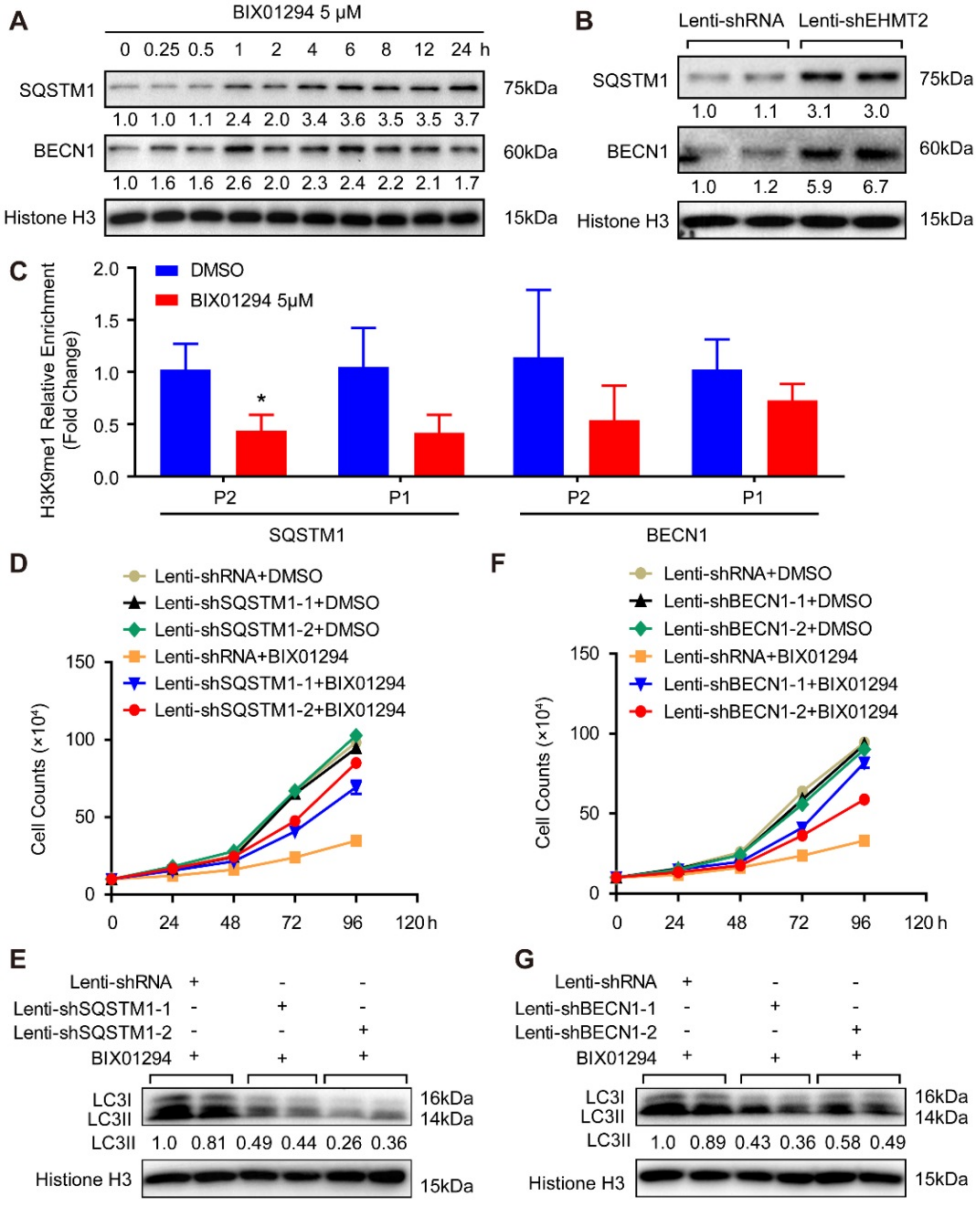
** EHMT2 directly inhibits SQSTM1 and BECN1 expression to suppress autophagic death of RAVSMCs. A and B.** SQSTM1 and BECN1 protein levels in RAVMSCs under **(A)** 5 μM of BIX01294 or DMSO or under **(B)** lenti-shRNA or lenti-shEHMT2 treatment were evaluate by western blot (n=4). Histone H3 serves as a loading control. **C.** ChIP-PCR detects the enrichment of H3K9me1 on the promoter of SQSTM1 and BECN1 in the RAVSMCs treated with 5 μM of BIX01294 or DMSO (n=3). *p<0.05 vs. DMSO. **D and F.** The growth curve of RAVSMCs treated with BIX01294 and lenti-shSQSTM1 **(D)** or treated with BIX01294 and lenti-shBECN1** (F)** (n=3). **E and G.** LC3 protein level was detected by western blot in RAVSMCs treated with indicated stimulations (n=4), and histone H3 serves as a loading control.

These results demonstrated that loss of EHMT2 function resulted in RAVSMC loss and that EHMT2 functioned as a brake during autophagy in RAVSMCs. To further explore the downstream molecules that mediate the effect of EHMT2 on autophagosome formation, we first tested the protein levels of autophagosome formation regulatory molecules, including ATG5, ATG7, ATG12, and ATG16L. Unexpectedly, none of the protein levels of these molecules was affected in the EHMT2-inhibited or knockdown group (Figure S2A and S2B). Previous studies revealed that SQSTM1 and BECN1 are the primary factors regulating autophagy initiation. Our results showed that both SQSTM1 and BECN1 protein levels considerably increased after BIX01294 stimulation for 1h (Figure 4A). Similarly, SQSTM1 and BECN1 showed a strikingly increasing trend in EHMT2 knockdown RAVSMCs (Figure 4B). To verify whether EHMT2 directly regulates SQSTM1 and BECN1 expression via H3K9me1, ChIP-PCR was performed by using H3K9me1 antibody. The results showed that H3K9me1 reduced its enrichment on the promoter of SQSTM1 and BECN1 after BIX01294 treatment in RAVSMCs (Figure 4C). As H3K9me1 is the suppressing histone marker, it reduced on the promoter of SQSTM1 and BECN1 indicated loss of the inhibition on these two genes. Next, we knocked down SQSTM1 and BECN1 in VSMCs separately and detected both protein levels. As shown in Figure S2C and S2D, SQSTM1 was successfully knocked down by lenti-shSQSTM1, and BECN1 was knocked down by lenti-shBECN1. Subsequently, we applied BIX01294 to RAVSMCs with or without SQSTM1 or BECN1 knockdown. Our results showed that SQSTM1 or BECN1 knockdown largely rescued the cell numbers of EHMT2-inhibited RAVSMCs, as evidenced by cell growth curve (Figure 4D and 4F) and cell images (Figure S2E and S2F). Furthermore, we found that the increased protein level of LC3II was also counteracted by SQSTM1 or BECN1 knockdown in EHMT2-inhibited RAVSMCs, indicating autophagy attenuated in these RAVSMCs (Figure 4E and 4G). Taken together, our results revealed that autophagy inhibition by SQSTM1 or BECN1 knockdown reversed the ACD induced by EHMT2 inhibition in RAVSMCs, which indicated that EHMT2 regulates the ACD of RAVSMCs by affecting SQSTM1- and BECN1-related autophagosome formation.

### EHMT2 inhibits autophagy in primary human aortic vascular smooth muscle cells

**Figure 5:**
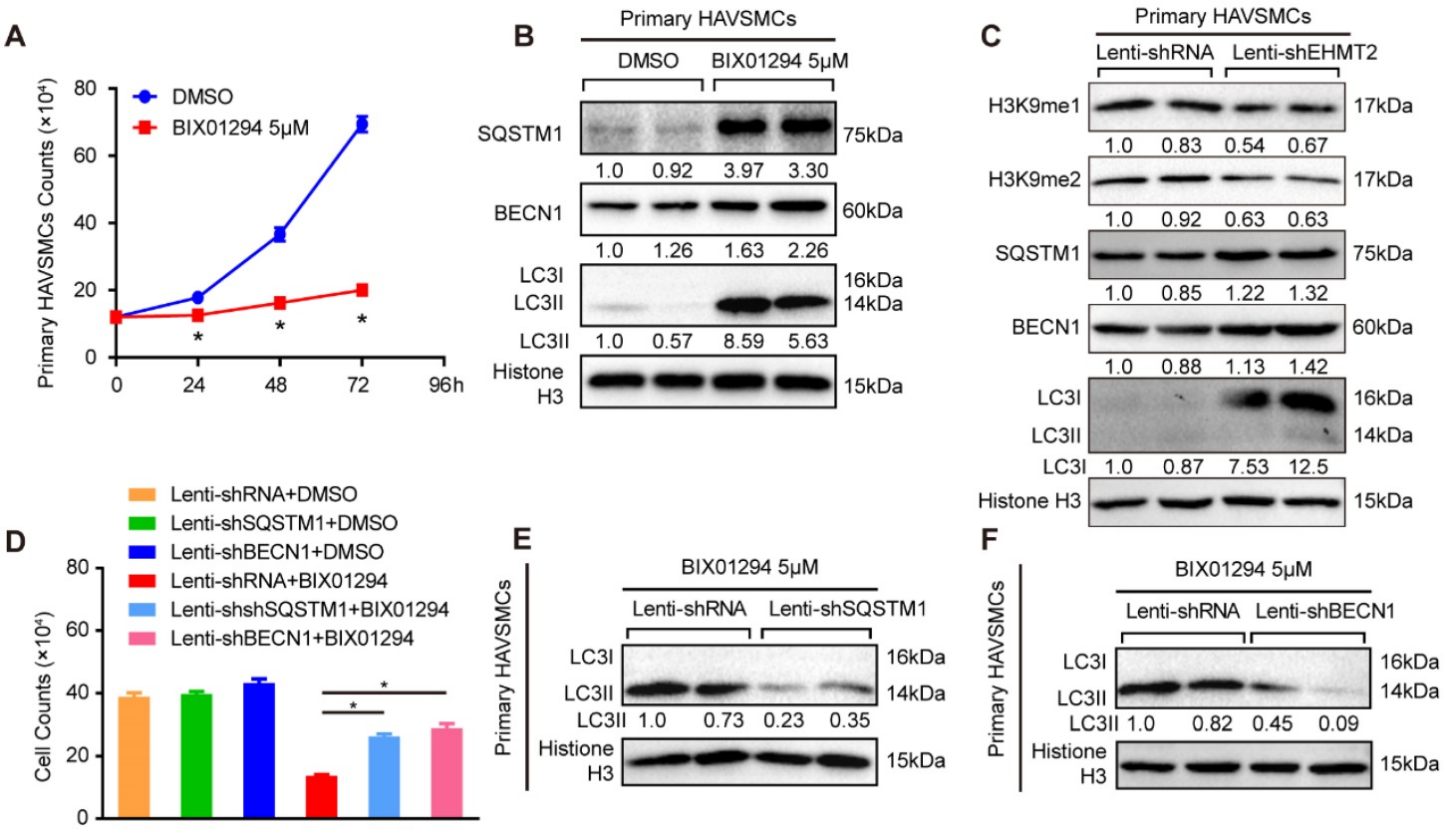
** EHMT2 suppresses autophagic death of primary HAVSMCs by regulating SQSTM1 and BECN1 expression. A.** The growth curve of primary HAVSMCs stimulated with BIX01294 for indicated times (n=3). *p<0.05 vs. DMSO. **B.** The protein levels of SQSTM1, BECN1, and LC3 were evaluated by western blot in primary HAVSMCs treated with BIX01294 or DMSO for 24h (n=4). **C.** H3K9me1, H3K9me2, SQSTM1, BECN1, and LC3 protein levels in primary HAVSMCs with EHMT2 knockdown or its counterparts (n=4). **D.** The growth curve of primary HAVSMCs treated with indicated stimulations (n=3). *p<0.05 vs. lenti-shRNA+ BIX01294. **E and F.** The LC3 protein levels were detected by western blot in primary HAVSMCs treated with indicated stimulations (n=4). Histone H3 serves as a loading control.

The present results revealed that EHMT2 plays a critical role in autophagy in RAVSMCs, which indicated that EHMT2 potentially participates in the pathophysiology of human aortic diseases. Thus, we made further efforts to verify the specific role of EHMT2 in primary HAVSMCs. As reported, we isolated primary HAVSMCs marked by α-SMA from the ascending aortas of heart transplantation donors (Figure S3A). We treated primary HAVSMCs with 5 μM of BIX01294 for the indicated times. EHMT2 was effectively inhibited, and H3K9me1/2 levels was correspondingly down-regulated (Figure S3B). Similar to the results in RAVSMCs, BIX01294 stimulation resulted in visible reductions in the cell numbers of primary HAVSMCs as early as 24 h, as shown in the cell images and growth curve (Figure 5A and Figure S3C). Moreover, the LC3II protein level increased in the EHMT2-inhibited group, accompanied by enhanced SQSTM1 and BECN1 protein levels, which indicated that EHMT2 inhibition enhanced the autophagy level in primary HAVSMCs (Figure 5B). However, BIX01294 treatment has no effect on primary HAVSMCs proliferation, as evidenced by comparable EdU positive cells between BIX01294 and DMSO group (Figure S3D). In HAVSMCs with EHMT2 knockdown (Figure S3E), the protein levels of H3K9me1 and H3K9me2 was reduced, while SQSTM1, BECN1, and LC3I levels were increased, which indicated EHMT2 deficiency enhanced autophagy activation (Figure 5C). However, similar percentage of the EdU positive cells between lenti-shRNA and lenti-shEHMT2 treated HAVSMCs indicated that EHMT2 loss of function has no effect on HAVSMCs proliferation (Figure S3F). Given that EHMT2 inhibits ACD via regulating SQSTM1 and BECN1 expression in RAVSMCs, we further verified this conclusion in HAVSMCs. Our data further validated that SQSTM1 or BECN1 knockdown could largely rescue the cell numbers reduced by BIX01294 treatment (Figure S3G-3J, Figure 5D), and reverse the LC3II protein level induced by BIX01294 in HAVSMCs (Figure 5E and 5F).

**Figure 6:**
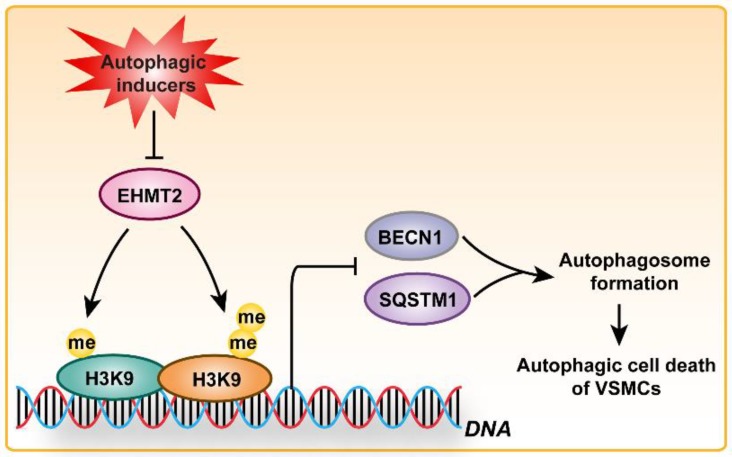
** Schematic summary.** Our results demonstrated that, under autophagic inducers treatment, EHMT2 protein level was downregulated. Inhibition of EHMT2 by BIX01294 or knockdown of EHMT2 by lenti-shEHMT2 facilitates autophagosome formation in VSMCs via directly increasing SQSTM1 and BECN1 expression, and then to accelerate autophagic cell death of VSMCs.

Altogether, the aforementioned results suggested that EHMT2 functions as a regulatory switch during VSMC loss via inhibiting autophagy, which is largely mediated by regulating SQSTM1 and BECN1 expression (Figure 6).

## Discussion

Changes in VSMC numbers or function plays a vital role in CVDs. Although EHMT2 has been observed to be highly associated with maintaining cellular homeostasis in several cancer lines, 25, 26 its epigenetic regulation in VSMCs has not yet been elucidated. Our data showed that inhibition of EHMT2 by BIX01294 or knockdown by shEHMT2 results in ACD of VSMCs. Interestingly, we observed that autophagic activation induced by rapamycin or nutrition deprivation was accompanied by a decrease in EHMT2 expression. Reduced EHMT2 accelerates the formation of autophagosomes via promoting SQSTM1 and BECN1 expression to induce ACD of VSMCs.

Autophagy is a highly conserved and multi-step cellular self-digestion process, which is an adaptive cellular process that occurs in response to different forms of stress by eliminating harmful cytosolic components, such as damaged organelles, long-lived or aggregated proteins, and pathogens, to generate amino acids and substrates for cellular metabolism to maintain cellular homeostasis.^27^ Further investigation of the upstream autophagy regulators, which provide a brake on the autophagy process, is crucial 28. Emerging evidence has shown that histone methylation and methyltransferase potentially act as a brake of autophagy.^28^-^31^ Shen* et al.* discovered that SMYD3 overexpression induced autophagic activetion in bladder cancer cells, which was associated with bladder cancer progression and poor prognosis 32. Shin *et al.* found that glucose starvation-induced autophagy was followed by increased H3R17 dimethylation levels resulting from co-activator-associated arginine methyltransferase 1 (CARM1) induction in the nucleus, and CARM1 was a transcriptional co-activator of autophagy-related and lysosomal genes through transcription factor EB (TFEB)-induced autophagic regulation 33. Our previous study identified EZH2 as a crucial component of autophagy in VSMCs. Our results showed that inhibition of EZH2 activity by UNC1999 or knockdown of EZH2 resulted in VSMC loss accompanied by increased expression of LC3B, ATG5 and ATG7 and formation of autophagosomes, while ATG5 or ATG7 knockdown resulted in autophagic inhibition and virtually rescued the VSMC loss induced by EZH2 inhibition or knockdown 8. Our present study showed that EHMT2 functions as an autophagic inhibitor in VSMCs, which supports and extends recent findings of the critical role of epigenetic regulation of autophagy.

Subsequently, we addressed the potential pattern of EHMT2 regulation of autophagy. EHMT2 is a nuclear histone lysine methyltransferase and a member of the Su(var)3-9 family, which mainly catalyzes histone H3 lysine 9 mono- and dimethylation, a reversible modification generally associated with transcriptional gene silencing 34. EHMT2 plays an important role in gene silencing and transcriptional repression 35. Our data showed that pharmacologic inhibition of EHMT2 or EHMT2 knockdown resulted in SQSTM1 and BECN1 expression activation. Furthermore, autophagic inhibition by either SQSTM1 or BECN1 knockdown could largely restore the growth of EHMT2-inhibited/knockdown VSMCs. More importantly, the protein level of LC3II was also reversed by SQSTM1 or BECN1 knockdown, which indicated that autophagy was reduced in these VSMCs. Recent studies reported that EHMT2 was enriched on the promoters of key genes during autophagy including LC3B, WD repeat domain phosphoinositide-interacting protein 1 (WIPI1), the diabetes- and obesity-regulated gene (DOR) in a c-Jun-dependent manner and BECN1, as well as the expression of B-cell lymphoma 19-kDa interacting protein (BNIP) 36, 37. Park *et al.* discovered that inhibition of EHMT2 reduced H3K9me2 and dissociated EHMT2 and H3K9me2 from the promoter of BECN1, and RNA polymerase II and nuclear factor kappa B (NF-κB) were recruited to the promoter in an ROS-dependent manner, resulting in transcriptional activation 38. These published results demonstrated that EHMT2 could regulate autophagy and autophagy-related molecules in cancer cell lines, but its role in the cardiovascular system, especially in VSMCs, remains unknown. Our results showed that EHMT2 manipulates autophagy in VSMCs by regulating H3K9me1 to directly suppress SQSTM1 and BECN1 expression.

Given that VSMCs is indispensable for aorta, and VSMCs death contributes to a variety of vascular diseases (*e.g.,* aortic dissection, aortic aneurysm and atherosclerosis), investigate and elucidate the mechanisms that regulates VSMCs death is urgently needed 39, 40. Autophagy is a “housekeeping” subcellular process that is important in maintaining normal cellular homeostasis and energy balance. It is still debated whether autophagy is a protective or harmful mechanism in vascular pathology. For example, our recently published results demonstrated that EZH2 loss of function facilitates autophagic death of VSMCs to aggravate aortic dissection, and we found that LC3, a hall mark of autophagy, is upregulated in the aortic samples of human aortic dissection patients 8. Importantly, another two independent research groups also verified this result 40, 41. In the present study, we demonstrated that EHMT2 inhibits autophagic death of VSMCs by directly regulating SQSTM1 and BECN1 expression. However, some other research group hold the opinion that autophagy is a protective process, because VSMC-specific deletion of autophagy related genes (*e.g.,* ATG5 and ATG7) usually impaired VSMC autophagy and increased cell death to affect atherosclerosis or dissecting aortic aneurysms 40, 42, 43. These studies further indicated that autophagy is a double-edged sword in VSMCs. Except for regulating autophagy, EHMT2 was recently reported to regulating inflammatory VSMC phenotype by dimethylating H3K9 (H3K9me2). H3K9me2 was enriched at a subset of inflammation-responsive gene promoters, including MMP3, MMP9, MMP12, and IL6, in VSMCs to affect post-injury neointima formation and atherosclerotic lesions 44. These studies indicated that EHMT2 may be involved in aortic dissection, aortic aneurysm and atherosclerosis via regulating autophagy or inflammatory response, but the exact roles and mechanisms of EHMT2 in these vasculopathy need further investigation.

## Conclusion

Taken together, our results indicate that inhibition of EHMT2 by BIX01294 or shEHMT2 induces autophagic cell death enhancement via regulating SQSTM1 or BECN1 expression, not only in RAVSMCs cell line but also in primary human VSMCs. Our data preliminarily showed that pharmacologic manipulation of EHMT2 could impact VSMC numbers and function and may be a potential treatment target for CVDs, such as aortic dissection.

## Supplementary Material

Supplementary figures.
