# Supplementary material for: EHMT2/G9a Inhibits Aortic Smooth Muscle Cell Death by Suppressing Autophagy Activation
Source: Int J Biol Sci. 2020 Feb 10;16(7):1252–63. doi: 10.7150/ijbs.38835 (PMC7053323; doi:10.7150/ijbs.38835)
Supplement: Supplementary file 1 — Supplementary figures. [file ijbsv16p1252s1.pdf]

Supplemental data

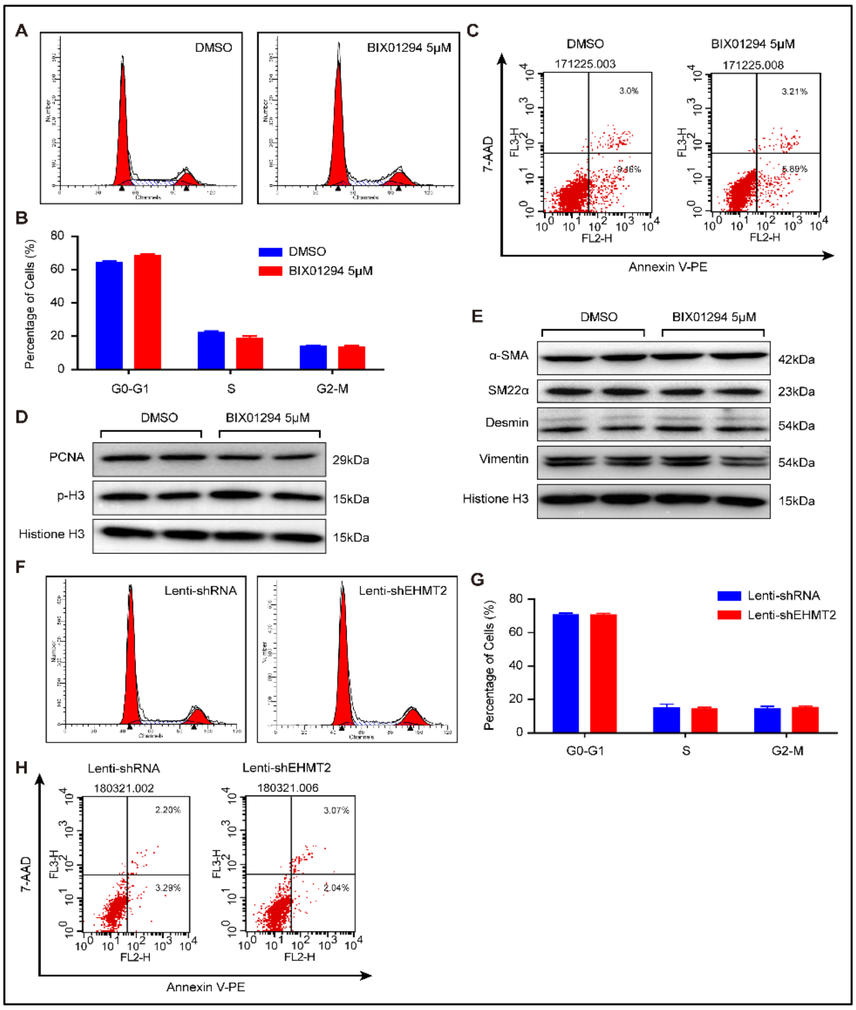

Figure S1. EHMT2 inhibition or knockdown has no effects on VSMCs proliferation and apoptosis. A and B. Cell cycle was measured by flow cytometry after treated with 5 μM BIX01294 for 48h, **A.** Representative results; **B.** The statistical results of cell ratio at different cell phases (n=4). **C.** The apoptosis was evaluated by PE Annexin V Apoptosis Detection Kit and detected by flow cytometry after treated with 5 μM BIX01294 for 48h (n=4). **D and E.** The markers of proliferation **(D)** and phenotype switching **(E)** were evaluated by western blot in RAVSMCs treated with 5 μM BIX01294 or DMSO for 24 h (n=4). **F and G.** Flow cytometry was performed to detect cell cycle of RAVSMCs treated with lenti-shRNA or lenti-EHMT2. **F.** Representative images of flow cytometry; **G.** The statistical results of cell ratio at different cell phases (n=4). **H.** Representative images of

RAVSMCs apoptosis measured by flow cytometry after infection with lenti-shRNA or lenti-EHMT2 (n=4).

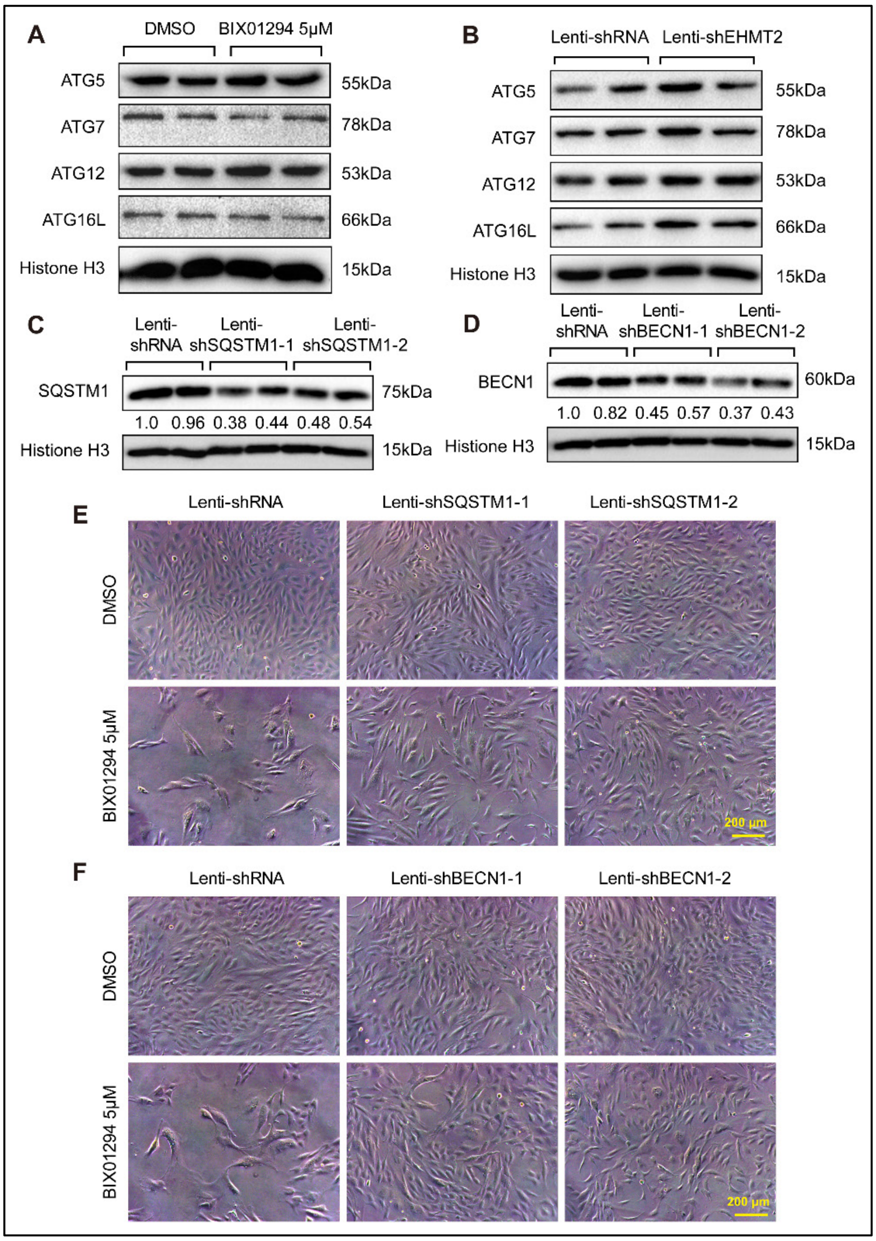

Figure S2. SQSTM1 and BECN1 knockdown largely rescued the cell numbers reduced by BIX01294 treatment. A and B. The protein levels of ATG5, ATG7, ATG12, ATG16L, and histone H3 were detected with western blot in RAVSMCs treated with **(A)** 5μM BIX01294 or DMSO or with **(B)** lenti-shRNA or lenti-shEHMT2 (n=4). **C and D.** The knockdown efficiency of SQSTM1 **(C)** and BECN1 **(D)** were evaluated by western blot (n=4), and histone H3 serves as a loading control. **E.** Cell images of RAVSMCs treated with BIX01294 and lenti-shSQSTM1 (n=4). **F.** Cell images of RAVSMCs treated with BIX01294 and lenti-shBECN1

(n=4).

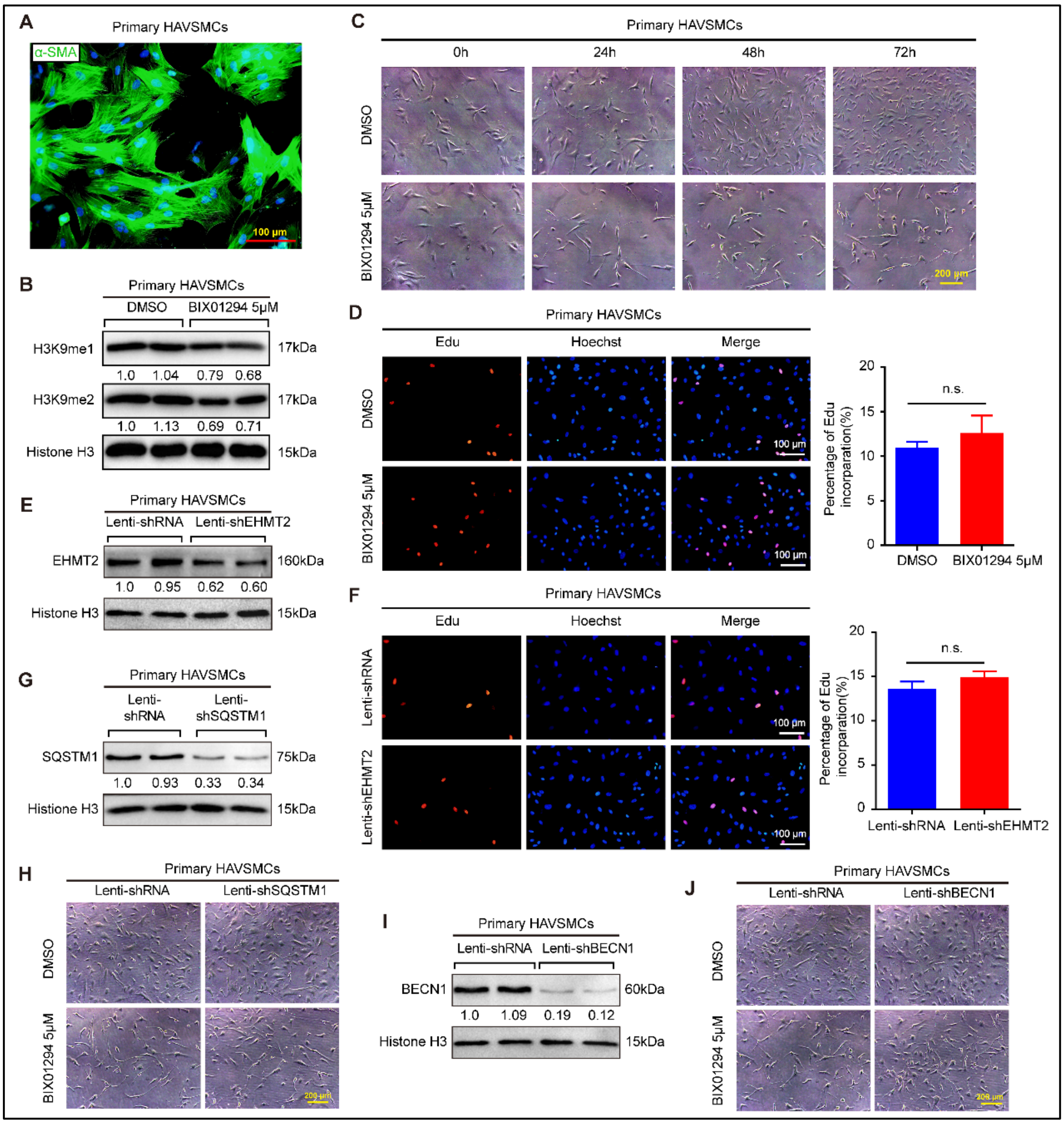

Figure S3. EHMT2 suppresses autophagy by regulating SQSTM1 and BECN1 expression in primary HAVSMCs. A. The primary HAVSMCs was identified by α-SMA fluorescence staining (n=3). **B.** Western blot was used to detect H3K9me1 and H3K9me2 protein levels in primary HAVSMCs treated with BIX01294 for 24h (n=4). **C.** The cell images of primary HAVSMCs stimulated with BIX01294 for indicated times (n=4). **D.** Edu incorporation assay was used to evaluate primary HAVSMCs proliferation after BIX01294 or DMSO treatment (n=4), the left: representative images of primary HAVSMCs, red: Edu, blue: nucleus; the right: statistical result, n.s. indicated no significance. **E.** The knockdown efficiency of EHMT2 in primary

HAVSMCs was evaluated by western blot (n=4), and histone H3 serves as a loading control. **F.** The proliferation was evaluated by Cell-LightTM Edu Apollo567 In Vitro kit and detected by fluorescent light in primary HAVSMCs after infected with lenti-shRNA or lenti-shEHMT2, the left: representative images of primary HAVSMCs, red: Edu, blue: nucleus; the right: statistical result, n.s. indicated no significance. **G.** The knockdown efficiency of SQSTM1 in primary HAVSMCs were evaluated by western blot (n=4), and histone H3 serves as a loading control. **H.** Cell images of primary HAVSMCs treated with BIX01294 and lenti-shSQSTM1 (n=4). **I.** The knockdown efficiency of BECN1 in primary HAVSMCs were evaluated by western blot (n=4), and histone H3 serves as a loading control. **J.** Cell images of primary HAVSMCs treated with BIX01294 and lenti-shBECN1 (n=4).
